# Mean arterial pressure trajectory with premature cardiovascular disease and all-cause mortality in young adults: the Kailuan prospective cohort study

**DOI:** 10.3389/fcvm.2023.1222995

**Published:** 2023-09-13

**Authors:** Zongshuang Song, Haiyan Zhao, Zhihao Wei, Wenliu Zhao, Yizhen Tan, Peng Yang, Shuohua Chen, YunTao Wu, Yun Li, Shouling Wu

**Affiliations:** ^1^ School of Public Health, North China University of Science and Technology, Tangshan, China; ^2^ Department of Cardiology, Kailuan General Hospital, Tangshan, China; ^3^ Department of Neurosurgery, Affiliated Hospital of North China University of Science and Technology, Tangshan, China

**Keywords:** blood pressure pattern, trajectory, cardiovascular diseases, all-cause mortality, young people

## Abstract

**Background:**

The association between mean arterial pressure (MAP) trajectory in young adults and risk of cardiovascular diseases (CVD) and all-cause mortality is not well-characterized. The objective of this study was to investigate the effects of different MAP trajectory on the risk of CVD and all-cause mortality among the young.

**Methods:**

In the Kailuan cohort study, 19,171 participants aged 18–40 years were enrolled without CVD (including myocardial infarction, stroke, atrial fibrillation and heart failure). The potential hybrid model was used to fit different trajectory patterns according to longitudinal changes of MAP. Hazard ratios and 95% confidence intervals for risk of CVD and all-cause mortality were analyzed using Cox proportional hazard regression models for participants with different trajectories.

**Results:**

Five distinct MAP trajectories were identified during 2006–2013. Each of the trajectories was labelled as low-stable, middle-stable, decreasing, increasing, or high-stable. With the low-stable trajectory group as the reference, the multivariate adjusted HR (95%CI) of CVD for the middle-stable, decreasing, increasing and high-stable groups were 2.49 (1.41–4.40), 5.18 (2.66–10.06), 5.91 (2.96–11.80) and 12.68 (6.30–25.51), respectively. The HR (95%CI) for all-cause deaths were 1.27 (0.84–1.94), 2.01 (1.14–3.55), 1.96 (1.04–4.3.72), and 3.28 (1.69–6.37), respectively.

**Conclusion:**

In young adults, MAP trajectories were associated with the risk of CVD or all-cause mortality and increasing MAP trajectories within the currently designated “normal” range may still increase the risk for CVD.

## 1. Introduction

Hypertension has been universally acknowledged as a paramount risk factor for cardiovascular diseases (CVD) and stroke (1, 2), rendering it the primary cause of worldwide disability and mortality (3). On an annual basis, high blood pressure accounts for over 9 million deaths, half of which are specifically attributed to CVD and stroke (4). Recent epidemiological studies have indicated a striking 50% surge in the incidence of CVD within young populations across the globe over the past decade, with a notable prevalence of 10%–15% occurring among adults aged 18–50 years (5). Despite this concerning trend, limited research has delved into the prospective impact of blood pressure on CVD within the confines of this youthful cohort.

Research data has revealed that approximately 70% of cardiovascular diseases (CVD) can be attributed to high blood pressure (6–9). Traditionally, the assessment of cardiovascular risk has relied on two primary components derived from the pressure waveform, one of which encompasses the stable aspect represented by mean arterial pressure (MAP) (10). While prior studies have predominantly concentrated on the individual impact of systolic blood pressure (SBP), diastolic blood pressure (DBP) (11), or pulse pressure (PP) (12) as independent risk factors, the potential influence of long-term MAP on CVD outcomes or mortality has received limited attention. Notably, MAP has demonstrated a superior predictive capability compared to SBP and DBP in forecasting the risk of CVD (13). Over the past two decades, the prognostic utility of MAP in predicting cardiovascular events and mortality has been extensively acknowledged. For instance, a substantial cohort study involving a vast population (*N* = 69,989; aged 40–69; mean follow-up time of 9.5 years) underscored the influential role of MAP (14), determined based on brachial arterial blood pressure measurements, in determining cardiovascular mortality.

However, a single blood pressure measurement may not be sufficient to predict the risk of long-term cardiovascular events. Changes in blood pressure over time were an principal factor that should be taken into account (15). In recent years, trajectories assessment has been widely used to reflect long-term patterns of blood pressure changes (11). Several studies have shown that different blood pressure trajectories were strongly associated with the occurrence of cardiovascular events (16, 17). However, few of these studies focused on MAP trajectories and young adults, which was why national guidelines for hypertension do not specifically recommend blood pressure management in young adults. To make up for these shortcomings, this study analyzed the effects of different MAP trajectories on the risk of early onset CVD and all-cause mortality in young adults based on the Kailuan study.

## 2. Methods

### 2.1. Study design and participants

The Kailuan study began in 2006, active and retired employees of Kailuan Group were enrolled and took the physical examination by Kailuan General Hospital and its 11 affiliated hospitals. After that, the cohort was followed up every two years. Information on the use of medications was obtained through questionnaires, and relevant physical examination data were also collected. Detailed data collection methods were described in our previous studies (18, 19).

In the current analyses, a total of 19,171 participants who were younger than 40 years old and without CVD in their first survey in 2006–2009 and did not have CVD until the baseline (the third survey was defined as the baseline) were included (the flow chart of participants selection was showed in Supplementary Figure S3). The study was approved by the Ethics Committee of Kailuan General Hospital and is in accordance with the Declaration of Helsinki.

### 2.2. Assessment of blood pressure

Face-to-face surveys were conducted by trained nurses and doctors in 2006 and during a biennial follow-up. BP in the left arm was measured using a mercury sphygmomanometer with an appropriately sized cuff, as per standard recommended procedures. The SBP is the point at which the first of two or more Korotkoff sounds are heard, and the disappearance of the Korotkoff sound is used to define the DBP. From 2014 onwards, the BP was measured using an electronic BP meter (HEM-8102A; Omron Limited, Dalian, China). After resting in a chair for at least 5 min, participants took at least two SBP and DBP readings every 5 min. BP was then measured again if the difference between the 2 measurements was ≥5 mmHg. MAP = 1/3SBP + 2/3DBP.

### 2.3. Follow up

MAP trajectories were determined based on the blood pressure from the first three surveys (referred as the trajectory period). Information of the third survey was defined as the baseline for the present study (Supplementary Figure S2). The outcome event was CVD or all-cause death. If the participants had multiple CVD, the first occurrence event was regarded as the outcome event. If no outcome event occurred, the follow-up ended on December 31, 2020. The physiological and biochemical indexes such as height, weight, blood glucose, and blood pressure were followed up by professionally trained doctors. Follow-up was performed every 2 years on average.

### 2.4. Assessment of CVD events and all-cause death

The outcome was CVD (including myocardial infarction, stroke, atrial fibrillation and heart failure). The diagnosis of CVD has been described in previous Kailuan study (20, 21). In brief, all participants were enrolled in the municipal social insurance agency and the registration form for cardiovascular discharge was universally covered among Kailuan study.

All suspected CVD events underwent medical record review by three experienced clinical referees who were blinded to the study design. According to the multi-country monitoring criteria for Trends and Determinants of CVD established by the World Health Organization (WHO), a diagnosis of occasional myocardial infarction is based on clinical symptoms, dynamic changes in myocardial enzyme or biomarker concentrations, and electrocardiogram results. Stroke diagnosis is based on symptoms and neuroimaging obtained through computed tomography (CT) or magnetic resonance imaging (MRI), with additional diagnostic reports provided in accordance with World Health Organization standards, as previously specified. The diagnosis of heart failure is based on criteria set by the European Society of Cardiology (22). Diagnostic criteria include clinical presentation and laboratory examination. All-cause death data were collected through provincial offices of Vital Statistics.

### 2.5. Covariates at baseline

Diabetes mellitus was defined as fasting blood glucose level ≥7.0 or <7.0 mmol/L but was taking hypoglycemic medications or with established diabetes. Hypertension is defined as having a systolic blood pressure of 140 mmHg or higher, a diastolic blood pressure of 90 mmHg or higher, a history of diagnosed hypertension, or taking medication for high blood pressure. Body mass index (BMI) = weight (kg)/height squared (m²). Smoking was defined as the consumption of at least one cigarette per day on average for a period of 1 year or more. Alcohol consumption was defined as an average daily intake of 100 ml liquor with over 50% alcohol content for a minimum duration of 1 year. Physical exercise was defined as engaging in physical activity at least three times per week. High-salt diet was defined as a daily salt intake of 10 g or more.

### 2.6. Statistical analyses

Statistical software SAS9.4 (SAS Institute, Cary, NC, USA) was used for data analysis. Each participant was followed up from the end of the baseline year until the date of CVD onset, death, loss of follow-up [of 1,820/19,171 (9.5%)], or end of follow-up (December 31, 2020), whichever came first.

Latent mixing modeling (PROC-TRAJ) was used to identify subgroups with similar latent MAP patterns, regardless of whether antihypertensive medications were used. Bayesian information criteria were used to evaluate model fit. We started a model with 5 trajectories, and then compared BIC's to BIC's with 4, 3, 2, and 1 trajectory, respectively. The model that identifies five trajectories was the best fit.

Cox proportional hazard regression model was used to analyze the effects of different MAP trajectories on the risk of CVD and all-cause mortality. Potential confounding factors [including age, sex, smoking status, alcohol consumption, physical activity, salt intake, use of glucose-lowering, blood pressure lowering, lipid-lowering medications, body mass index (BMI), estimated glomerular filtration rate (eGFR), triglyceride (TG) concentration, high density lipoprotein (HDL-c), low density lipoprotein (LDL-c), and high sensitivity C-reactive protein (hs-CRP)] were included for correction. The proportional-hazards assumption was satisfied. Kaplan-Meier method was used to calculate the cumulative incidence of cardiovascular events and all-cause death in groups with different MAP trajectories, and Log-rank test was used for comparison among groups. All statistical tests were 2-sided, and *P* < 0.05 was regarded as significant.

#### 2.6.1. Sensitivity analysis

Because taking antihypertensive, glucose-lowering, and lipid-lowering medications might have some influence on the results, repeated Cox proportional risk regression analysis was conducted to analyze the effect of different MAP trajectories on the risk of CVD and all-cause mortality after excluded the population taking medications during the trajectory period or during the follow-up. To examine whether the potential association between MAP trajectory and the risk of CVD or all-cause mortality could be explained by either MAP status, MAP at first survey and at baseline was further corrected.

## 3. Results

**Figure 1:**
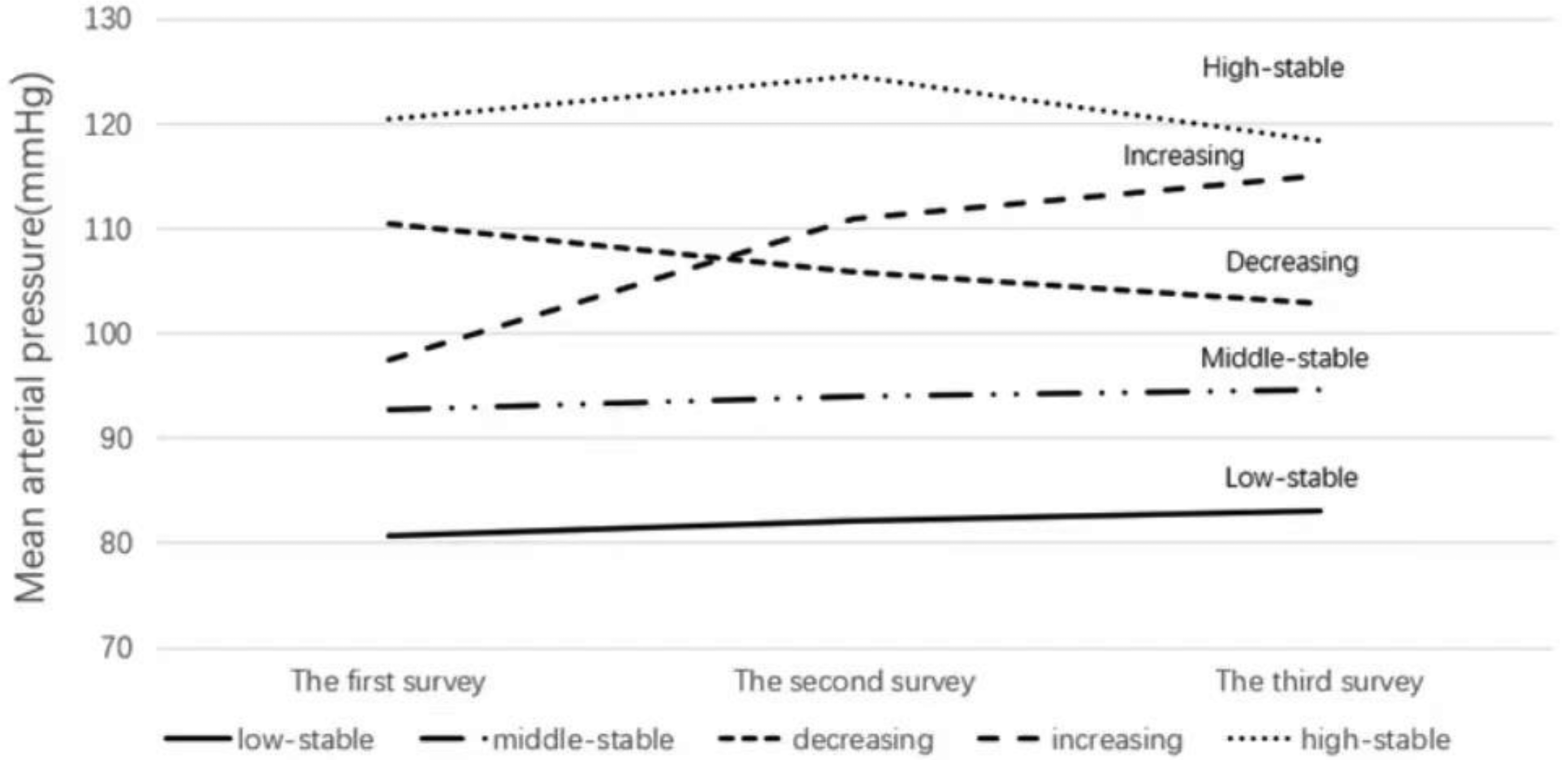
Mean arterial pressure trajectory patterns.

Of the 19,171 subjects in the study, 14,825 were male, accounting for 77.3% of the subjects (Table 1). The baseline mean age was (35.9 ± 5.5) years. During the trajectory period, five different trajectories were identified. MAP was consistently stable at around 120 mmHg in 2.2% (*n* = 405) of participants (referred as the “high stable mode”), around 90–110 mmHg in 53.7% (*n* = 10,893) of participants (referred as the “middle stable mode”), around 80–90 mmHg in 27.3% (*n* = 4,973) of participants (referred as the “low stable mode”). 5.8% (*n* = 920) of participants had a MAP of about 100 mmHg in the first survey, which increased to about 110 mmHg during the trajectory period (referred as the “increasing mode”); 11% (*n* = 1,980) of participants had an average arterial pressure of approximately 110 mmHg at their first survey and continued to decline to 100 mmHg during the trajectory period (referred to as the “decreasing mode”) (Figure 1).

**Table 1:** Characteristics of 19,171 participants according to MAP trajectories.

Items	Total (*N* = 19,171)	Low-stable (*N* = 4,973)	Middle-stable (*N* = 10,893)	Decreasing (*N* = 1,980)	Increasing (*N* = 920)	High-stable (*N* = 405)	*P*-value^*^
Age, year	35.9 ± 5.5	34.8 ± 5.6	35.8 ± 5.5	37.9 ± 4.8	38.0 ± 4.9	38.7 ± 4.7	<.0001
Male, *N* (%)	14,825 (77.3)	2,488 (50.0)	9,223 (84.7)	1,873 (94.6)	851 (92.5)	390 (96.3)	<.0001
Current smoker, *N* (%)	7,131 (37.2)	1,143 (23.0)	4,408 (40.5)	904 (45.7)	450 (48.9)	226 (55.8)	<.0001
Current drinker, *N* (%)	8,264 (43.1)	1,382 (27.8)	5,083 (46.7)	1,001 (50.6)	553 (60.1)	245 (60.5)	<.0001
Education level, *N* (%)							<.0001
≤junior high school	7,457 (38.9)	1,562 (31.4)	4,218 (38.7)	954 (48.2)	478 (52.0)	245 (60.5)	
≥senior high school	11,714 (61.1)	3,411 (68.6)	6,775 (61.3)	1,026 (51.8)	442 (48.0)	160 (39.5)	
Salt intake >=10 g/d, *N* (%)	2,008 (10.5)	441 (8.9)	1,189 (10.9)	208 (10.5)	125 (13.6)	45 (11.1)	0.0001
Physical activity, *N* (%)	12,871 (67.1)	3,225 (64.9)	7,387 (67.8)	1,378 (69.6)	596 (64.8)	285 (70.4)	0.0002
BMI, kg/m^2^	24.8 ± 3.7	22.7 ± 3.0	25.1 ± 3.5	26.7 ± 3.6	27.1 ± 3.7	27.7 ± 3.9	<.0001
FBG, mmol/L	5.2 ± 0.8	5.0 ± 0.6	5.2 ± 0.8	5.5 ± 1.0	5.5 ± 1.0	5.7 ± 1.4	<.0001
LDL-C, mmol/L	2.5 ± 0.7	2.3 ± 0.7	2.5 ± 0.7	2.6 ± 0.7	2.7 ± 0.7	2.6 ± 0.8	<.0001
HDL-C, mmol/L	1.5 ± 0.4	1.5 ± 0.4	1.4 ± 0.4	1.4 ± 0.4	1.4 ± 0.4	1.5 ± 0.4	<.0001
TC, mmol/L	4.7 (4.1–5.2)	4.4 (4.0–5.0)	4.7 (4.1–5.2)	4.9 (4.2–5.4)	5.0 (4.4–5.6)	5.0 (4.5–5.7)	<.0001
TG, mmol/L	1.3 (0.9–2.0)	1.0 (0.7–1.4)	1.4 (1.0–2.1)	1.6 (1.1–2.6)	1.9 (1.3–3.0)	1.9 (1.3–2.9)	<.0001
hs-CRP, mg/L	1.0 (0.4–2.1)	0.7 (0.4–1.6)	1.0 (0.4–2.2)	1.2 (0.2–2.2)	1.2 (0.7–2.6)	1.6 (0.8–3.6)	<.0001
eGFR, ml/min	108.0 (87.5–116.7)	111.3 (93.5–119.1)	107.7 (87.1–116.3)	98.4 (79.2–113.0)	107.8 (91.9–114.8)	99.5 (83.8–112.3)	<.0001
Antihypertensive treatment, *N* (%)	1,346 (7.0)	48 (1.0)	424 (3.9)	398 (20.1)	263 (28.6)	213 (52.6)	<.0001
Antidiabetic treatment, *N* (%)	334 (1.7)	63 (1.3)	188 (1.7)	44 (2.2)	28 (3.0)	11 (2.7)	<.0001
Lipid-lowering treatment, *N* (%)	179 (0.9)	20 (0.4)	76 (0.7)	45 (2.3)	18 (2.0)	20 (4.9)	<.0001
Sbp in baseline	120.7 ± 14.7	106.7 ± 9.6	121.5 ± 9.4	132.6 ± 9.7	146.5 ± 11.3	153.4 ± 14.5	<.0001
Dbp in baseline	80.7 ± 10.1	71.1 ± 6.8	81.2 ± 6.3	88.6 ± 6.9	99.6 ± 7.0	103.2 ± 9.7	<.0001
Map in baseline	94.1 ± 11.1	83.0 ± 7.0	94.7 ± 6.5	103.3 ± 6.8	115.2 ± 7.1	120.0 ± 10.3	<.0001
Sbp in first survey	118.9 ± 15.2	104.3 ± 9.2	119.4 ± 9.6	141.7 ± 10.6	126.4 ± 9.5	153.8 ± 13.9	<.0001
Dbp in first survey	78.9 ± 10.4	68.9 ± 6.5	79.3 ± 6.4	94.7 ± 6..6	83.1 ± 6.3	103.8 ± 9.2	<.0001
Map in first survey	92.2 ± 11.5	80.7 ± 6.7	92.7 ± 6.6	110.4 ± 6.5	97.5 ± 6.4	120.5 ± 9.6	<.0001
Hypertension	7,143 (37.3)	78 (1.6)	3,767 (34.6)	1,973 (99.6)	920 (100)	405 (100)	<.0001
Diabetes	1,048 (0.9)	97 (2.0)	562 (5.2)	238 (12.0)	94 (10.2)	57 (14.1)	<.0001

Data are presented as *n* (%), mean ± SD or median (interquartile range) according to variable category.

BMI, body mass index; FBG, fasting blood glucose; LDL-C, low-density lipoprotein cholesterol; HDL-C, high-density lipoprotein cholesterol; hs-CRP, high-sensitivity C reactive protein; TG, triglyceride; TC, total cholesterol; SBP, systolic blood pressure; DBP, diastole blood pressure; MAP, mean arterial pressure.

* *P*, comparison of baseline characteristics among different MAP groups.

During a mean follow-up period of 9.67 ± 1.44 years, 310 subjects (1.62%) had cardiovascular diseases, including 201 stroke, 49 myocardial infarction, 46 heart failure, and 25 atrial fibrillation. The mean age of occurring the cardiovascular diseases was 45.49 ± 4.88 years. The incidence density was 0.31/1,000, 1.22/1,000, 3.98/1,000, 5.16/1,000 and 11.95/1,000 person years for the low-stable, middle-stable, decreasing, increasing and high-stable groups, respectively (Table 2). There were 267 all-cause deaths, and the incidence density was 0.70/1,000, 1.22/1,000, 2.79/1,000, 2.80/1,000 and 6.12/1,000 person years for the low-stable, middle-stable, decreasing, increasing and high-stable groups, respectively (Table 3). There were statistically significant differences for the incidence of CVD or all-cause death across the different trajectory components (Log-rank test, *P* < 0.05, Supplementary Figure S1).

**Table 2:** Adjusted hazard ratios and 95% confidence intervals for risks of CVD according to MAP trajectories.

	Low-stable	Middle-stable	Decreasing	Increasing	High-stable
Total CVD					
*n*/*N*	15/4,973	129/10,893	76/1,980	45/920	45/405
Incidence rate, per 1,000 person-years	0.31	1.22	3.98	5.16	11.95
Model 1	Ref.	3.06 (1.77–5.29)	8.07 (4.54–14.37)	10.27 (5.60–18.81)	22.35 (12.15–41.11)
Model 2	Ref.	2.85 (1.64–4.96)	7.13 (3.97–12.81)	8.69 (4.69–16.13)	18.11 (9.69–33.82)
Model 3	Ref.	2.49 (1.41–4.40)	5.18 (2.66–10.06)	5.91 (2.96–11.80)	12.68 (6.30–25.51)
Stroke					
*n*/*N*	10/4,973	83/10,893	49/1,980	29/920	30/405
Incidence rate, per 1,000 person-years	0.21	0.78	2.55	3.29	7.89
Model 1	Ref.	3.01 (1.53–5.90)	7.86 (0.86–15.97)	9.90 (4.69–20.87)	22.17 (10.49–45.86)
Model 2	Ref.	2.89 (1.47–5.71)	7.33 (3.56–15.07)	8.91 (4.16–19.08)	19.28 (8.952–41.52)
Model 3	Ref.	2.50 (1.24–5.05)	5.13 (2.26–11.66)	5.94 (2.52–13.95)	13.02 (5.50–30.81)
Non-stroke					
*n*/*N*	5/4,973	46/10,893	27/1,980	16/920	15/405
Incidence rate, per 1,000 person-years	0.104	0.43	1.39	1.79	3.82
Model 1	Ref.	2.79 (1.08–7.19)	6.56 (2.43–17.69)	9.39 (3.32–26.59)	15.54 (5.41–44.66)
Model 2	Ref.	2.42 (0.93–6.28)	5.07 (1.84–13.94)	7.19 (2.47–20.88)	11.41 (3.86–33.69)
Model 3	Ref.	2.12 (0.78–5.68)	3.89 (1.24–12.18)	4.62 (1.41–15.15)	8.28 (2.48–27.59)

Model 1: Adjusted for age and gender.

Model 2: Adjusted for age, gender, smoking, drinking, education level, salt status, physical activity and BMI.

Model 3: Adjusted for all the variables in model 2 and TG, LDL-C, HDL-C, hs-CRP, eGFR, hypertension and diabetes.

**Table 3:** Adjusted hazard ratios and 95% confidence intervals for risks of all-cause deaths according to MAP trajectories.

	Low-stable	Middle-stable	Decreasing	Increasing	High-stable
*n*/*N*	34/4,973	130/10,893	54/1,980	25/920	24/405
Incidence rate, per 1,000 person-years	0.71	1.22	2.79	2.80	6.12
Model 1	Ref.	1.29 (0.87–1.91)	2.28 (1.45–3.60)	2.24 (1.31–3.84)	3.84 (2.19–6.76)
Model 2	Ref.	1.35 (0.91–2.01)	2.45 (1.53–3.91)	2.41 (1.39–4.19)	4.09 (2.29–7.31)
Model 3	Ref.	1.28 (0.84–1.94)	2.01 (1.14–3.51)	1.96 (1.04–3.72)	3.28 (1.69–6.37)

Model 1: Adjusted for age and gender.

Model 2: Adjusted for age, gender, smoking, drinking, education level, salt status, physical activity and BMI.

Model 3: Adjusted for all the variables in model 2 and TG, LDL-C, HDL-C, hs-CRP, eGFR, hypertension and diabetes.

Different MAP trajectories were grouped as independent variables, and the occurrence of CVD or all-cause deaths as dependent variables, respectively. Cox proportional hazard regression results showed that compared with participants in the low-stability group, The HR (95%CI) of total CVD were 2.48 (1.40–4.40), 5.18 (2.66–10.06), 5.91 (2.96–11.80) and 12.68 (6.30–25.51) for participants in the middle-stable, decreasing, increasing and high-stable groups, respectively (Table 2). The HR (95%CI) for all-cause deaths were 1.27 (0.83–1.94), 2.01 (1.14–3.55), 1.96 (1.04–3.72), and 3.28 (1.69–6.37), respectively (Table 3). After classifying CVD into stroke and non-stroke (including myocardial infarction, atrial fibrillation and heart failure), similar results were observed for participants with decreasing, increasing or high-stable MAP trajectories. However, for participants with middle-stable MAP trajectory, the association was observed only for stroke, not for non-stroke (Table 2).

To eliminate the impact of medication intake on the outcomes, the Cox proportional hazard regression model was repeated after excluding the patients who took antihypertensive, hypoglycemic, or lipid lowering medications during the trajectory or follow-up period (Supplementary Table S1). The results were consistent with the main analysis. Further adjustment for baseline MAP and first survey MAP generated similar results (Table 4).

**Table 4:** Association between risks of diseases and MAP trajectories after adjusted MAP in the first survey or in baseline.

	Low-stable	Middle-stable	Decreasing	Increasing	High-stable
Total CVD					
Model 1	Ref.	1.88 (1.04–3.39)	2.62 (1.22–5.65)	4.25 (2.08–8.71)	4.77 (1.94–11.72)
Model 2	Ref.	2.11 (1.14–3.88)	4.24 (2.05–8.78)	4.19 (1.83–9.63)	7.93 (3.29–19.06)
All-cause deaths					
Model 1	Ref.	0.92 (0.58–1.45)	0.91 (0.45–1.85)	1.33 (0.68–2.61)	1.05 (0.42–2.61)
Model 2	Ref.	1.17 (0.72–1.91)	1.65 (0.85–3.21)	1.63 (0.71–3.75)	2.10 (0.79–5.59)
Stroke					
Model 1	Ref.	2.01 (0.97–4.17)	3.01 (1.16–7.78)	4.59 (1.89–11.13)	6.04 (1.99–18.33)
Model 2	Ref.	2.21 (1.03–4.72)	4.65 (1.88–11.46)	4.15 (1.48–11.67)	7.85 (2.64–23.31)
Non-stroke					
Model 1	Ref.	1.49 (0.54–4.16)	1.69 (0.46–6.31)	3.17 (0.93–10.75)	2.58 (0.56–11.90)
Model 2	Ref.	1.94 (0.69–5.42)	3.61 (1.06–12.34)	4.69 (1.17–18.86)	8.67 (1.97–38.19)

Model 1: Adjusted for age, gender, smoking, drinking, education level, salt status, physical activity, BMI, TG, DL-C, HDL-C, TC, hs-CRP, eGFR, hypertension, diabetes and MAP in the first survey.

Model 2: Adjusted for age, gender, smoking, drinking, education level, salt status, physical activity, BMI, TG, LDL-C, HDL-C, hs-CRP, eGFR, hypertension, diabetes and MAP in baseline.

## 4. Discussion

In the present study, we followed 19,171 young participants (less than 40 years) for about 9 years and observed that the MAP trajectories were associated with the risk of CVD and all-cause death in young adults. Compared to participants with low stable MAP trajectory, the CVD risk was 2.49–12.68 folds higher for those with middle-stable, decreasing, increasing or high-stable MAP trajectories, and the all-cause death risk was 2.01–3.28 folds higher for those with decreasing, increasing or high-stable MAP trajectories. There was a lack of specific studies on MAP changes and disease risk in previous studies. However, some studies showed that the single blood pressure value was associated with the cardiovascular disease risk, which was similar to our results (13, 23). The findings of our study suggested that monitoring blood pressure trajectory should be helpful in identifying individuals with high risk of CVD or death, and preventing the occurrence of CVD and death in young population.

The trajectory approach takes into account mean, variability and direction of variation, so it was better in understanding and illustrating the evolution of disease risk (24). In the present study, the participants with increasing trajectory had higher risk of CVD or all-cause death, despite their MAP at the first survey was lower than those with decreasing trajectory. The results confirmed that using a single blood pressure value to predict cardiovascular risk might misclassify the population at risk, whereas long-term blood pressure changes can predict disease risk more effectively.

Our findings revealed that individuals with consistently elevated mean arterial pressure (MAP) falling within the range of 90–100 mmHg, which is considered within the normal MAP range, exhibited a significantly higher risk of cardiovascular disease (CVD) compared to those with a consistently lower MAP below 90 mmHg. These results align with Sesso's study (13), which demonstrated an increased risk of CVD in individuals younger than 60 years old with a MAP exceeding 88 mmHg. Consequently, implementing more rigorous blood pressure control strategies may prove to be more effective in preventing CVD among young adults. However, determining the optimal blood pressure target for preventing cardiovascular events in the young population remains unclear. The SPRINT trial compared the occurrence of CVD in a non-diabetic cohort following either an enhanced blood pressure control strategy or a standard blood pressure control strategy. The study found that the enhanced blood pressure control strategy resulted in a lower incidence of major cardiovascular events and overall mortality (25). These findings, along with our study results, collectively underscore the importance of strengthening blood pressure control measures to reduce the risk of CVD in the young population.

Our study further analyzed the association of MAP trajectory and baseline MAP with CVD. The results showed that the influence of baseline MAP on CVD risk disappeared after the MAP trajectory was brought into the same model, but the MAP trajectory was still associated with CVD. It suggested that the association between MAP trajectory and CVD was stronger than baseline MAP. Although there was no previous study to compare the strength of the association between MAP trajectory or baseline MAP and CVD, Portegies et al. found a stronger correlation between blood pressure trajectory and stroke than blood pressure at baseline level (17), which supported our results to some extent. It was also found that the association between MAP trajectory and CVD was independent of blood pressure at the first survey, which strongly supports the necessity of long-term blood pressure monitoring to reduce the risk of CVD. However, the results were not similar for all-cause death in our study. Baseline MAP seems to have a greater impact on the risk of death than MAP trajectories, which was consistent with other studies (23).

We also observed that the risks of CVD or death in participants with early and remained elevated blood pressure (HR (95%CI) were 12.68 (6.30–25.51) for CVD and 3.28 (1.69–6.37) for all-cause death) were higher than that in participants with late elevated blood pressure (HR (95%CI) were 5.91 (2.96–11.80) for CVD and 1.96 (1.04–3.72) for all-cause death). It suggested that the risks of CVD and all-cause mortality in people with persistently high levels of MAP were higher than those in people with MAP rising to high levels in a short period. This was consistent with the conclusions of a study based on data from the Atherosclerosis Risk in Communities Study (26), which found that subjects whose SBP remained above 140 mmHg had a higher CVD death risk than subjects whose SBP rose sharply from pre-hypertensive levels to more than 140 mmHg. Our results showed that for those with early elevated MAP, although lowering MAP might decrease the risk of CVD or all-cause death, the risks were still higher even their elevated MAP decreased to normal range (HR (95%CI) were 5.18 (2.66–10.06) for CVD and 2.01 (1.14–3.55) for all-cause death). On the one hand, the results demonstrated the importance of lowing blood pressure as early as possible. On the other hand, the results emphasized the importance of primary prevention in disease controlling, that was, early prevent the occurrence of diseases.

Previous studies have shown that longitudinal changes in blood pressure were negatively correlated with ankle-brachial index and positively correlated with pulse wave velocity, and suggested a relationship between changes in blood pressure and impaired vascular function (27). Long-term exposure to higher blood pressure may lead to endothelial cell damage, which may alter the interaction between blood cells and endothelial cells, eventually leading to local thrombosis and ischemic lesions, leading to cardiovascular and cerebrovascular diseases (28, 29). Fibrous necrosis is considered a potential risk factor for CVD, resulting in lacunar infarction through focal stenosis and occlusion. Degenerative changes in endothelial cells and smooth muscle cells may lead to aneurysm formation and cerebral hemorrhage. High blood pressure also speeds up the atherogenic process, increasing the likelihood of brain damage associated with narrowing and embolization caused by large extracranial blood vessels (30). In addition, some known risk factors for CVD, such as left ventricular hypertrophy, are also associated with long-term elevated blood pressure. Therefore, a longer pattern of blood pressure monitoring may better reflect these pathophysiological changes. MAP acts as a major driving force for vital organ perfusion is associated with CVD and all-cause mortality (31). More detailed studies are needed to elucidate the role of MAP longitudinal changes in the development of mortality and CVD and the related mechanisms to help refine our understanding and guidance on optimal blood pressure management in young adults.

We found that the MAP trajectory pattern was associated with the risk of CVD or all-cause death in young adults and was independent of baseline MAP. Therefore, early controlling of blood pressure and long-term monitoring of blood pressure can effectively reduce the risk of CVD or all-cause death, thereby reducing the financial burden of CVD on patients’ quality of life and on families and society. In addition, young individuals with CVD might lose their ability to work prematurely. Blood pressure control should be strengthened to reduce the occurrence of CVD or death in young people. In all, our results had important clinical and public health implications in disease controlling for young population.

### 4.1. Limitations

This study is subject to certain limitations that should be considered. Firstly, all participants included in our study were employed by Kailuan Study in Tangshan, China. Therefore, the identified trajectories may not be generalized to other populations. However, the homogeneity of our cohort can help reduce confounding factors such as racial and healthcare differences, thereby enhancing content validity. Secondly, our cohort had a relatively small representation of women, and we did not perform gender stratification to assess potential differences between blood pressure trajectory and CVD risk. This limits our ability to fully understand gender-specific associations. Lastly, it is important to note that the study population consisted of young individuals who are generally at lower risk for CVD. Consequently, the number of CVD events observed was relatively small, which may impact the level of certainty in our findings.

## 5. Conclusion

Our study showed that MAP trajectories were associated with the risk of CVD or all-cause mortality and increasing MAP trajectories within the currently designated “normal” range may still increase the risk for CVD. Monitoring blood pressure trajectory can provide an important way to identify people at high risk and decrease the occurrence of CVD or all-cause death in young population.

## Data Availability

The original contributions presented in the study are included in the article/**Supplementary Material**. Requests to access these datasets should be directed to Yun Li, liyun8022@163.com.
