# Supplementary material for: Mean arterial pressure trajectory with premature cardiovascular disease and all-cause mortality in young adults: the Kailuan prospective cohort study
Source: Front Cardiovasc Med. 2023 Sep 13;10:1222995. doi: 10.3389/fcvm.2023.1222995 (PMC10525694; doi:10.3389/fcvm.2023.1222995)
Supplement: Supplementary file 1 [file Datasheet1.pdf]

Supplementary Material

1 Supplementary Figures and Tables

Table s1. Hazard ratios and 95% confidence intervals for disease risk after excluding medicine users

	Low-stable	Middle-stable	Decreasing	Increasing	High-stable
**Total CVD**					
Sensitivity analysis1*	Ref.	2.29(1.28-4.07)	4.35(2.18-8.70)	4.71(2.25-9.87)	9.16(4.08-20.54)
Sensitivity analysis2*	Ref.	2.21(1.17-4.17)	4.48(2.04-9.88)	5.27(2.28-12.16)	10.10(4.11-24.81)
**All-cause mortality**					
Sensitivity analysis1*	Ref.	1.30(0.84-2.00)	2.08(1.14-3.79)	1.56(0.74-3.28)	3.67(1.69-7.99)
Sensitivity analysis2*	Ref.	1.32(0.85-2.03)	2.27(1.25-4.12)	2.64(1.36-5.12)	4.26(2.10-8.64)
**Non-stroke**					
Sensitivity analysis1*	Ref.	1.89(0.70 -5.13)	2.78(0.83-9.24)	3.89(1.10-13.72)	4.29(1.02-18.01)
Sensitivity analysis2*	Ref.	2.12(0.68-6.60)	3.49(0.83-14.66)	4.45(0.96-20.58)	9.19(1.89-44.74)
**Stroke**					
Sensitivity analysis1*	Ref.	2.32(1.14-4.71)	4.57(1.95-10.71)	4.59(1.83-11.48)	10.07(3.76-26.95)
Sensitivity analysis2*	Ref.	2.15(1.01-4.62)	4.55(1.77-11.65)	5.22(1.91-14.22)	9.17(3.06-27.43)

Sensitivity analysis1：Excluding 1346 participants who used anti-hypertensive during trajectory;

Sensitivity analysis2：Excluding 1846 participants who using anti-hypertensive, hypoglycemic or lipid-lowering medications during follow-up;

**Adjusted for age, gender, smoking, drinking, education level, salt status and physical activity, BMI, TG, LDL-C, HDL-C, hs-CRP, eGFR, hypertension and diabetes.*

Table s2. Baseline characteristics of included and excluded participants

	Total (N=22615)	Excluded (N=3444)	Included (N=19171)	P Value
Age, year	35.7±5.4	34.3±4.7	35.9±5.5	<.0001
Male, N (%)	16920(74.8)	2095(60.8)	14825(77.3)	<.0001
Current smoker, N (%)	8268(36.5)	1137(33.0)	7131(37.2)	<.0001
Current drinker, N (%)	9506(42.0)	1242(36.1)	8264(43.1)	<.0001
Education level, N (%)				<.0001
≤junior school high	9009(39.8)	1552(45.1)	7457(38.9)	<.0001
≥senior school high	13606(60.2)	1892(54.9)	11714(61.1)	<.0001
Salt intake>=10g/d, N (%)	2309(10.2)	301(8.7)	2008(10.5)	0.0083
Physical activity, N (%)	15756(69.7)	2885(83.8)	12871(67.1)	<.0001
BMI, kg/m²	24.7±3.7	24.1±3.4	24.8±3.7	<.0001
FBG, mmol/L	5.2±0.8	5.2±1.0	5.2±0.8	<.0001
LDL-C, mmol/L	2.5±0.7	2.5±0.7	2.5±0.7	<.0001
HDL-C, mmol/L	1.5±0.4	1.5±0.5	1.5±0.4	<.0001
TC, mmol/L	4.7(4.1-5.2)	4.8(4.1-5.2)	4.7(4.1-5.2)	0.1463
TG, mmol/L	1.3(0.9-2.0)	1.2(0.9- 1.7)	1.3(0.9-2.0)	<.0001
hs-CRP, mg/L	1.0(0.4-2.1)	1. 1(0.5-2.4)	1.0(0.4-2.1)	<.0001
eGFR, mL/min	107.3(85.6- 116.6)	98.1(75.3- 115.9)	108.0(87.5- 116.7)	<.0001
Hypertension	7755(34.3)	612(17.8)	7143(37.3)	<.0001
Diabetes	1164(5.1)	116(3.4)	1048(5.5)	<.0001

Footnotes: BMI, body mass index; FBG, fasting blood glucose; LDL-C, low-density lipoprotein cholesterol; HDL-C, high-density lipoprotein cholesterol; hs-CRP, high-sensitivity C reactive protein; TG, triglyceride; TC, total cholesterol. Data are presented as n (%), mean ± SD or median (interquartile range) according to variable category.

1.1 Supplementary Figures

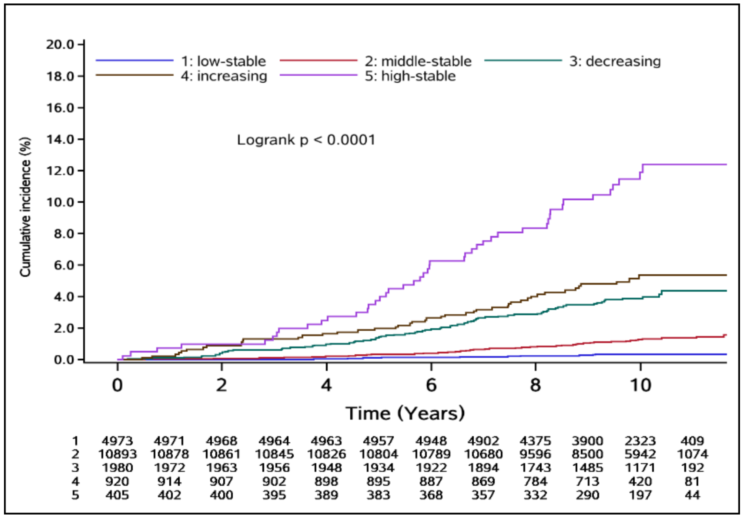

A.CVD

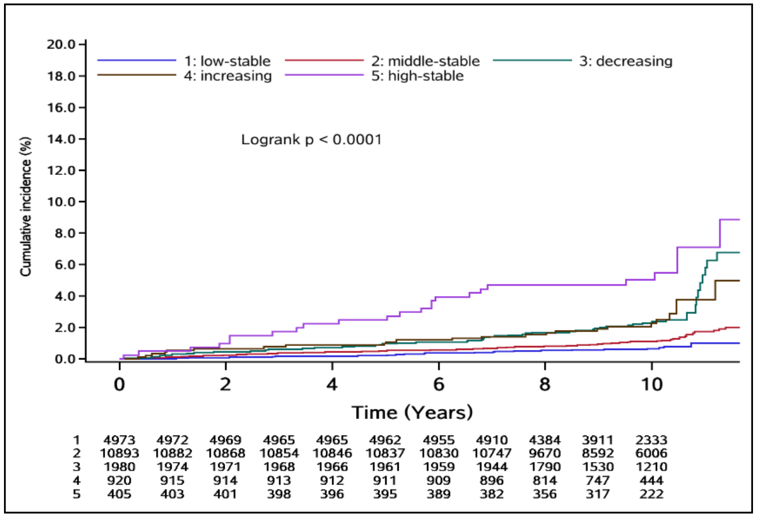

B. all-cause mortality

Figure s1. Cumulative incidence of different MAP trace group.

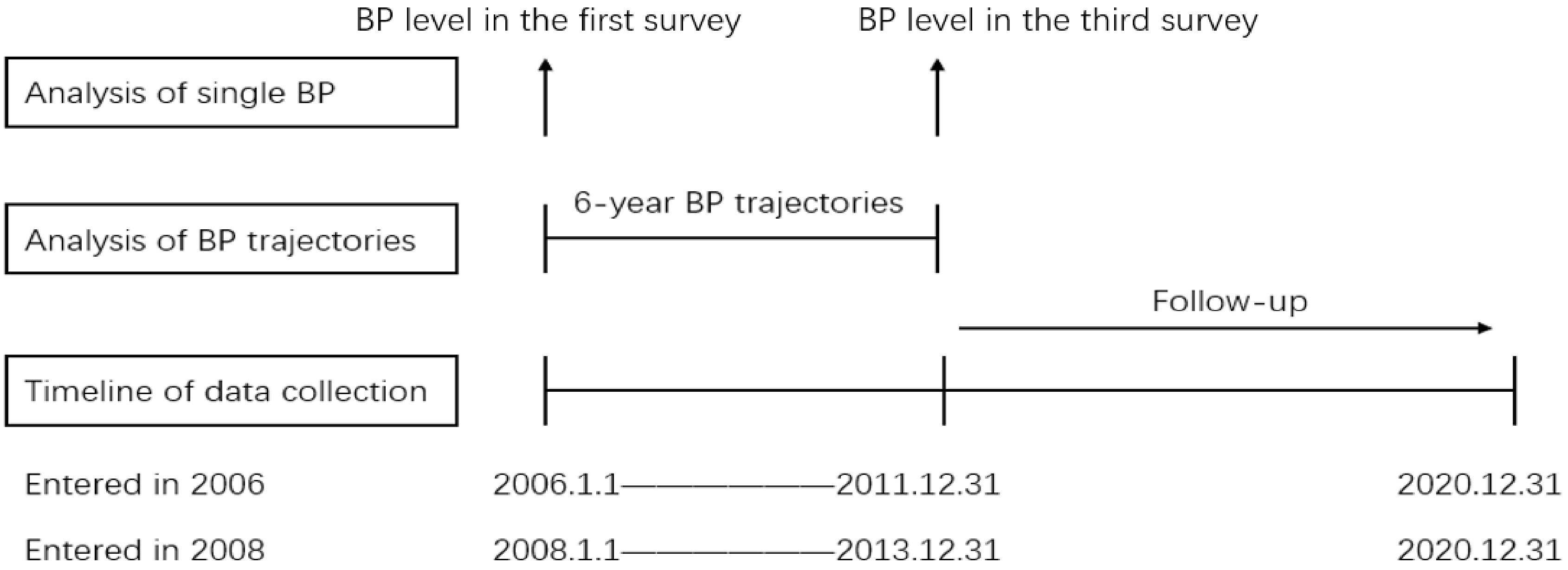

Figure s2. Overview of the study design.

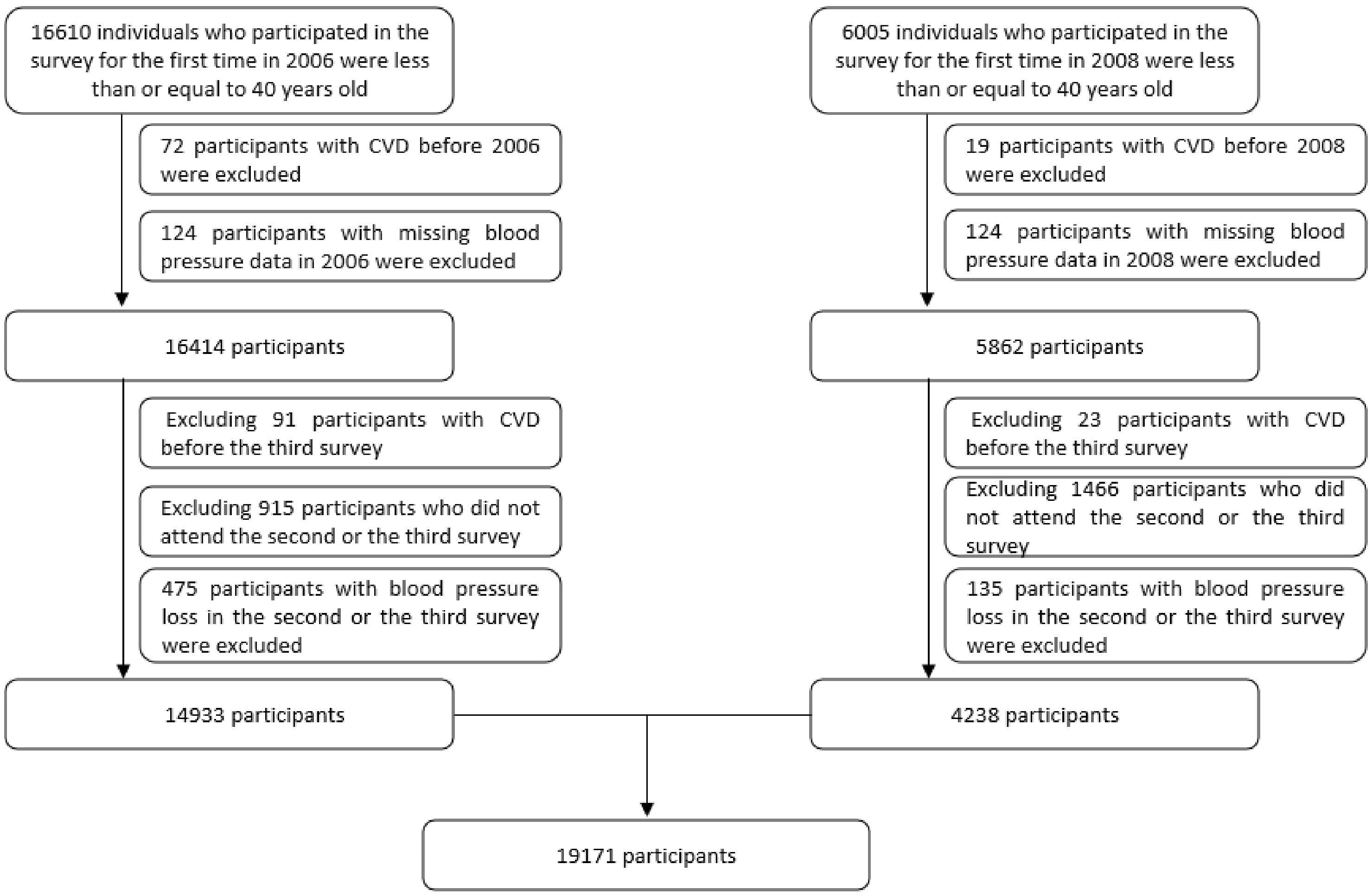

Figure s3. Flow chart of subject selection process.
